# Preoperative Hypertension

**DOI:** 10.1007/s40140-018-0248-7

**Published:** 2018-02-08

**Authors:** Simon James Howell

**Affiliations:** Leeds Institute of Biomedical and Clinical Sciences, University of Leeds, Clinical Sciences Building, St James’s University Hospital, Leeds, LS9 7TF UK

**Keywords:** Hypertension, Blood pressure, Perioperative medicine, Antihypertensive

## Abstract

**Purpose of Review:**

This review will examine the implications for perioperative management of new hypertension guidelines and place these in the context of findings from recent large observational studies.

**Recent Findings:**

Recent hypertension guidelines highlight the role of ambulatory blood pressure measurement with the implication that isolated preoperative blood pressure measurements are of limited value. There is emerging evidence from large observational studies that both preoperative and intraoperative hypotension are associated with increased risk. It is not clear if this is a particular concern for hypertensive patients.

**Summary:**

Assessment of the hypertensive surgical patient should include blood pressure measurements taken using the correct technique. Preoperative blood pressures of less than 180/100 mmHg are not grounds for deferring surgery in the absence of active comorbid disease. Evidence to guide the perioperative management of patients with higher pressures is scanty and decisions should be made on a case-by-case basis.

## Introduction

Hypertension is common. In 2011, 31% of men and 28% of women in the UK were classified as hypertensive, defined as having a blood pressure greater than 140/90 mmHg [1]. The burden of hypertension is greater in older people with 72.6% of people aged over 75 years considered to be hypertensive. Raised blood pressure is associated with life-threatening comorbidities including ischaemic heart disease, heart failure, renal impairment, and cerebrovascular disease. Whilst the incidence of these complications can be reduced by effective blood pressure management, achieving good blood pressure control at a population level remains challenging. Of the 29.5% of adults considered to be hypertensive in 2011, only 10.4% had adequate blood pressure control, with 6.4% having inadequate blood pressure control despite treatment and 12.7% with untreated hypertension. Hypertension is relevant to the anaesthetist for a number of reasons including the associated target organ damage, the possibility of the patient having a serious condition causing secondary hypertension, the question of the best management of blood pressure in hypertensives in the perioperative period, and the broader responsibility on all healthcare staff to ensure that newly diagnosed and poorly controlled hypertension is adequately treated.

## The Definition and Diagnosis of Hypertension

Threshold blood pressures for the diagnosis of hypertension are based on the association between a given blood pressure and comorbid disease such as ischaemic heart disease or renal failure. There is a continuum of risk, higher blood pressures being associated with a higher incidence of complications. Severe hypertension, for example a systolic blood pressure of greater than 180 mmHg is uncommon but carries very considerable risks for the patient. Lesser degrees of raised blood pressure such as a blood pressure of 140–150 mmHg systolic are much more common and, whilst they carry less risk for the individual patient, are responsible for a great deal of the population burden of cardiovascular disease. The definition and categorisation of hypertension rests on striking a balance between the prevention of such cardiovascular disease and the requirement to treat a large proportion the population with antihypertensive medications [2]. Various national guidelines identify cut-off values for hypertension at similar but not identical blood pressure levels. In the USA, the Eighth Joint National Committee on the Prevention, Detection, Evaluation, and Treatment of High Blood Pressure (JNC8) recommends treatment for raised blood pressure for patients aged 60 years or older without diabetes or chronic kidney disease with a goal of reducing the blood pressure to less than 150 mmHg systolic and 90 mmHg diastolic [3]. For patients aged under 60 years, the targets are a systolic blood pressure of less than 140 mmHg and a diastolic pressure of less than 90 mmHg. These targets are less demanding than in the previous JNC7 guideline which recommended treating blood pressure to target of less than 140/90 mmHg and to less than 130/80 mmHg in patients with diabetes or renal disease [4]. The UK National Institute for Health Care and Clinical Excellence (NICE) 2011 guidance defines normotension as a blood pressure of less than 140/90 mmHg when the measurements are made in the clinical setting [5••]. The guidance categorises hypertension as stage I (a clinic blood pressure of 140/90 to 160/90 mmHg or an ambulatory blood pressure of 135/85 to 150/95 mmHg) and stage II (a clinic blood pressure of 160/90 to 180/109 mmHg or an ambulatory blood pressure of greater than 150/95 mmHg). The most recent NICE guidelines added a category of severe hypertension with a clinic systolic blood pressure of 180 mmHg or greater or a diastolic blood pressure of 110 mmHg or greater. Whilst a diagnosis of stage I or stage II hypertension requires multiple measurements on different occasions, treatment may be initiated at once in patients with severe hypertension. These guidelines also place emphasis on the use of blood pressure monitoring outside of the clinic. The guidance identifies ambulatory monitoring as the preferred method for BP recording to diagnose hypertension with home blood pressure readings taken by the patient as the second choice if ambulatory monitoring is not available. In contrast, the 2013 European Society of Hypertension and European Society of Hypertension/European Society of Cardiology guidance lists ambulatory and home blood pressure monitoring as additional tests to be used if required [6].

Whichever guidelines are being applied, it is essential that appropriate technique is used when measuring the blood pressure. The patient should be sitting quietly with the arm supported at heart level. The patient should not be engaging in conversation. An appropriate size cuff should be used. If an automated device is used, it should conform to the appropriate national standards [7]. The pulse should be palpated before the reading is taken and manual measurements made if the patient is in an irregular rhythm such as atrial fibrillation. If the BP is elevated, a second reading should be taken at least a minute after the first reading. If there is a marked discrepancy between these two blood pressure readings, a third reading should be taken again after a suitable interval [6]. These standards are often not met in the preassessment clinic or on the ward before surgery and if a patient being assessed for surgery is found to have elevated blood pressure, it is appropriate to repeat the blood pressure readings ensuring that the correct technique is used.

Blood pressures recorded in patients coming to hospital for elective surgery are often elevated. It would not be appropriate to diagnose new or poorly controlled hypertension on the basis of blood pressure recordings from the preassessment clinic. However, the effect of the medical setting is related to the true blood pressure with “white-coat” hypertension being seen in 55% of people with grade 1 hypertension but only about 10% of people with severe hypertension [8]. Patients who have markedly elevated blood pressures in the preassessment clinic are more likely to have persistently raised blood pressure and should be referred to their general practitioner or community physician for further follow-up.

Hypertension is of concern because of the extensive damage that it can cause throughout the body. Target organ damage in the heart may be manifest as left ventricular hypertrophy, diastolic dysfunction, heart failure, microvascular disease, and atherosclerotic coronary artery disease [2]. The brain may be affected by lacuna infarcts and impaired cognition. Hypertension is a major cause of stroke and severe hypertension may produce hypertensive encephalopathy. The vascular damage caused by hypertension is directly visible in the eye as hypertensive retinopathy. The kidneys may be affected by glomerular injury, glomerulosclerosis, renal tubular ischaemia, and end-stage renal failure. The wider circulation may be affected by peripheral arterial disease which in the worst cases can lead to critical limb ischaemia and limb loss. The assessment of the presence and severity of target organ damage is an important aspect of the preoperative assessment of the hypertensive patient. The presence of such damage is an indicator of the severity of hypertension. The patient who presents with both raised blood pressure and its sequelae is unlikely to have white coat hypertension. Investigations of the patient presenting with raised blood pressure should include full blood count; serum sodium, potassium, and creatinine; plasma glucose; urine testing for microalbuminuria; and a 12-lead ECG [5••]. Comprehensive assessment of the hypertensive patient also includes measurement of serum uric acid, cholesterol, and high-density and low-density lipoprotein. Performing these additional tests is helpful if the patient is to be referred on for further assessment of their blood pressure.

## The Aetiology of Hypertension

Blood pressure is regulated by a number of integrated cardiovascular control systems. The immediate control of blood pressure over the course of 10 to 20 s is through changes in heart rate and vascular tone in response to blood pressure changes sensed by the carotid and aortic baroreceptors. Control of blood pressure over minutes to hours is through the regulation of circulating volume. Low-pressure receptors in the atria and great veins regulate the release of antidiuretic hormone and atrial natriuretic factor. The long-term control of the “set-point” around which an individual’s blood pressure is regulated is through the renin-angiotensin-aldosterone system (RAAS) which controls natriuresis and through this, diuresis [9]. The RAAS has a high gain. That is to say small changes in blood pressure produce a vigorous response and return the blood pressure to its set point.

In primary hypertension, the set point for blood pressure is elevated. Primary hypertension was previously known as essential hypertension and has been renamed to better reflect our incomplete understanding of the mechanisms of this change. It is thought that primary hypertension is due to the interaction of genetic and environmental factors [10]. Genetic variation in the sodium homeostasis and the RAAS is thought to have a significant role. Environmental factors include alcohol consumption, psychological stress, high sodium intake, low potassium intake, and low calcium intake.

It has been proposed plasma renin activity (PRA) may be useful to guide the treatment of hypertensive patients. This approach suggests that patients with low-renin, volume-dependant hypertension (PRA < 0.65 ng/ml^−1^/h^−1^) should be treated with an “anti-volume” drug such as a diuretic whereas those with high-renin vasoconstriction hypertension (PRA > 0.65 ng ml^−1^ h^−1^) should be managed with an antagonist of the renin-angiotensin system such as an ACE inhibitor [11]. These recommendations have not been adopted into the various national and international guidelines on hypertension but PRA-guided therapy remains an area of active research. The thesis carries the implication for intraoperative care that the subset of patients with “pure” high-renin hypertension will have marked arteriolar vasoconstriction and may be particularly sensitive to the vasodilating effects of anaesthetic agents.

Approximately 95% of patients presenting with raised blood pressure will be considered to have primary hypertension. In a small number of patients, it will be possible to identify an underlying pathology. These patients are considered to have secondary hypertension and are a particular concern because many of the conditions underlying secondary hypertension have pernicious effects both on the long-term health of the patient and the well-being of the patient during surgery and anaesthesia. Younger patients with raised blood pressure may have secondary hypertension and any hypertension in an individual aged less than 20 years or raised blood pressure requiring treatment in an individual younger than 30 years should prompt a search for the causes of secondary hypertension. A sudden onset or worsening of hypertension or hypotension resistant to treatment with three or more drugs should prompt consideration of secondary hypertension.

The clinician should be alert for the symptoms and signs of diseases that can cause raised blood pressure. Elevated serum creatinine, the presence of proteinuria or haematuria, polycystic kidneys identified on imaging, or the presence of a murmur or angiographic evidence of renal artery stenosis should lead to consideration of renal hypertension. Delayed or absent femoral pulses and an audible murmur may indicate the presence of aortic coarctation. The hypertensive patient who has hypokalaemia with an increased plasma sodium may have Conn’s syndrome due to a mineralocorticoid-secreting tumour. The signs of Cushing’s syndrome should prompt consideration of this diagnosis. The patient who has the stigmata of neurofibromatosis may have an associated pheochromocytoma.

## The Treatment of Hypertension

The treatment of hypertension is directed towards lowering blood pressure in order to prevent or reduce end organ damage and the risk of premature death or disability. The management of the patient should be set in the context of a broader estimation of long-term cardiovascular risk. Where there is a history of previous cardiovascular disease, the patient should be receiving appropriate cardiovascular secondary prevention such as antiplatelet agents and statins [12, 13].

Care should include lifestyle advice to the patient on issues including regular exercise, weight loss, avoiding excess alcohol consumption, and strategies to reduce psychological stress where possible. In some cases, these changes may be sufficient to reduce the blood pressure; however, many patients will require pharmacological treatment.

The commonly used antihypertensive drugs may be divided into three broad classes, those that increase salt and water excretion, those that act on the RAAS, and vasodilators that increase salt and water excretion through mechanisms other than the RAAS [14, 15].

### Drugs Acting on the RAAS

Drugs which act upon the RAAS reduce the production of angiotensin II or inhibit its action. Angiotensin II is produced by the action of angiotensin-converting enzyme on angiotensin I. Angiotensin II has multiple actions. It acts on AT_1_ receptors increasing arteriolar tone and systemic vascular resistance. It increases sympathetic nervous system activation. Its hormonal effects include increased pituitary secretion of antidiuretic hormone and ACTH and increased secretion of aldosterone by the adrenal cortex. Drugs which interfere with these actions reduce systemic vascular resistance and salt and water retention, thus lowering blood pressure.

The angiotensin-converting enzyme inhibitors (ACEI) act by reducing the conversion of angiotensin I to angiotensin II in the lungs. This class of drugs includes the oral drugs captopril, enalapril, ramipril, perindopril, and lisinopril. Enalaprilat is the active form of enalapril and is administered intravenously. ACEI are first-line agents for the treatment of primary hypertension in patients aged less than 55 years.

The angiotensin receptor antagonist (ARA) drugs act on AT_1_ receptors and antagonise the action of angiotensin II. Losartan and valsartan are competitive antagonists whilst candesartan and telmisartan bind irreversibly with the AT_1_ receptor. The ARAs do not inhibit bradykinin metabolism and because of this, ARAs are less likely than ACEI to cause a chronic cough. They also have the potential merit of blocking the action of angiotensin II produced by pathways other than angiotensin-converting enzyme such as that are produced by the action of chymase in the kidney.

Both ACEI and ARAs can produce relative hypovolaemia and there are reports of an increased incidence of hypotension with induction of anaesthesia with both these classes of drug. This is said to be particularly marked with a combination of ARAs and diuretics [16•, 17]. Many units have local guidelines recommending the omission of one or two doses of ACEI or ARAs prior to induction of anaesthesia. Doing so does not appear to be associated with an increased risk of postoperative hypertension [18].

Aliskiren is a direct renin inhibitor which is administered orally and has a place in the treatment of hypertension refractory to management with other medications. It has a long elimination half-life of 24 to 40 h and there are concerns that it may be associated with refractory hypotension following induction of anaesthesia. However, the data to support this are limited [19].

Beta-blockers are no longer considered to be first-line antihypertensive agents unless also being administered for other indications such as secondary prevention following myocardial infarction. They act to inhibit catecholamines at G protein-coupled β-adenoreceptors and reduce blood pressure through multiple mechanisms. The blockade of cardiac β-1 receptors reduces heart rate and beta-blockade also reduces myocardial contractility. Blockade of β-receptors in the juxtaglomerular apparatus reduces renin secretion and thus salt and water retention.

### Vasodilator Antihypertensive Drugs

#### Calcium Channel Blockers

Calcium channel blockers (CCBs) are first-line treatment for primary hypertension in patients aged over 55 years and in black patients of African or Caribbean origin. CCBs act on L-type calcium channels in vascular smooth muscle and the heart. Those with a greater affinity for the myocardium, for example verapamil and diltiazem, have a role in the management of tachyarrhythmias and angina. CCBs with a particular affinity for vascular smooth muscle, such as nifedipine, produce vasodilatation and are favoured for the management of hypertension. It should be noted that nifedipine can cause significant vasodilatation with a reflex tachycardia. There has long been the concern that sublingual nifedipine can produce dramatic reductions in blood pressure and myocardial ischaemia in elderly patients [20]. CCBs are generally continued through the perioperative period. There is little or no evidence to support initiating CCBs in the perioperative period for myocardial protection unless there is a clear medical indication for their prescription.

#### Alpha-1 Receptor Antagonists

Alpha-1 blockers act at post-ganglionic α-1 G_q_ protein-coupled receptors causing smooth muscle relaxation and vasodilatation. Selective alpha-1 blockers such as doxazosin are used in the management of hypertension that is refractory to treatment with first-line drugs alone. Nonselective alpha blockers act at both α-1 and α-2 receptors. Blockade of presynaptic alpha-2 receptors increases norepinephrine release and may cause tachycardia. However, nonselective alpha blockers such as phenoxybenzamine and phentolamine have a place in the management of pheochromocytoma. Labetalol acts as an antagonist at α-1 receptors and is also a nonselective β-receptor antagonist; thus, it lowers blood pressure by causing vasodilatation and can counteract the reflex tachycardia that may result from a fall in blood pressure produced by α-1 receptor blockade and vasodilation.

### Diuretics

Thiazide (bendrofluazide, hydrochlorothiazide) and thiazide-like diuretics (chlorthalidone, indapamide) are used to treat hypertension in patients intolerant of CCBs and in those who have or are at risk of heart failure. They also are widely prescribed as second-line drugs in patients who have not responded to initial pharmacological treatment. They inhibit sodium and chloride reabsorption in the proximal part of the distal tubular and so produce diuresis. They also act directly on vascular smooth muscle opening calcium-activated potassium channels and reducing systemic vascular resistance. The side effects of thiazide diuretics are well known and have direct relevance to perioperative management. Patients taking these drugs may suffer from hypokalaemia, hyponatraemia, hypercalcaemia, hypomagnesaemia, hyperuricaemia, hypercholesterolaemia, hypoglycaemia, and hypokalaemia alkalosis.

Aldosterone antagonists such as spironolactone are used as fourth-line agents in the treatment of hypertension. The loop diuretics have a place in the management of resistant hypertension in patients with heart failure or chronic kidney disease.

### Other Antihypertensive Drugs

Direct acting vasodilators include hydralazine and minoxidil. Vasodilator drugs cause side effects including headache, fluid retention, and oedema and are generally poorly tolerated. However, hydralazine has a place in the management of pre-eclampsia.

Centrally acting drugs such as clonidine, methyldopa, and monondinine are associated with significant adverse effects and are reserved for refractory hypertension. Again, methyldopa has a specific place for the management of hypertension in pregnancy.

## The Association Between Preoperative Blood Pressure and Perioperative Risk

Historically, concerns about the care of the hypertensive patient have focused on the perceived risks of anaesthesia and surgery and the appropriateness of deferring surgery to allow the blood pressure to be treated and reduced.

The risks of anaesthesia and surgery in hypertensive patients were highlighted in a case series published in 1929 which described the outcomes of a series of patients with cardiac disease who underwent anaesthesia and surgery at the Massachusetts General Hospital between 1917 and 1927 [21]. Twenty-four of the 75 patients with atherosclerotic or hypertensive heart disease died, one half succumbing to heart failure. The ensuing decades brought an increasing understanding of the health risks posed by high blood pressure and the introduction of the first effective treatments for hypertension. A series of studies published in the early 1970s suggested that untreated hypertensives undergoing anaesthesia and surgery displayed greater cardiovascular instability and were at greater risk of myocardial ischaemia than those receiving treatment for hypertension [22–24]. On the basis of these findings, it became a recommended practice that patients with poorly controlled or untreated hypertension should have their blood pressure brought under control before anaesthesia and surgery. These studies were conducted on a relatively small number of patients. The definition of hypertension has been revised several times since this work was done with the blood pressure thresholds that define hypertension being revised downwards repeatedly. Hence, it is not appropriate to use the findings of these studies to inform practice in patients with moderately raised blood pressure, that is with blood pressures consistent with stage 1 or stage 2 hypertension [25].

Studies conducted over the past 20 years suggest that mild-to-moderate preoperative hypertension is not a major risk factor for complications [26–29]. Whilst a number of studies have shown a univariate association between increased blood pressure and perioperative cardiac complications, this association tends to disappear when adjustment is made for major cardiac risk factors such as heart failure and previous myocardial infarction. (It should of course be noted that these represent the sequelae of hypertension in many patients.) The American College of Cardiology/American Heart Association Guidelines on Perioperative Evaluation and Care for Non-Cardiac Surgery do not identify hypertension as a risk factor that should lead to the deferring or cancelling surgery [30]. The European Society of Cardiology/European Society of Anaesthesiology guidelines take a similar position [31]. This is not to say that preoperative hypertension should be disregarded completely as a cause for concern in perioperative management. There is evidence that these patients are more prone to perioperative myocardial ischaemia [32]. The European guidelines highlight the fact that patients may display increased cardiovascular instability particularly at induction of anaesthesia. The anaesthetist should be aware of these concerns and manage the patient accordingly.

Evidence is scanty regarding the management of patients who are present for surgery and are found to have markedly raised blood pressure on repeated measurement. Patients presenting in primary care with severe hypertension would be considered for the immediate initiation of antihypertensive treatment without further clinic visits. The European guidelines suggest that delaying surgery for further assessment may be considered in patients who are found to have blood pressure readings consistently greater than 180 mmHg systolic or 110 mmHg diastolic. [31] This decision should be made on a case-by-case basis taking into account the relative considerations of cardiovascular risk and the potential harm from delaying surgery.

Data are now emerging from large databases that may be relevant to this discussion. A recent analysis of a large UK primary care database of over quarter of a million patients found an association between postoperative mortality and elevated diastolic pressure [33••]. Elevated diastolic, but not systolic, pressure was found to be associated with increased postoperative mortality with the association beginning at diastolic blood pressures of greater than 90 mmHg. These findings are striking given the fact that in the nonsurgical setting, diastolic hypertension is a more potent cardiovascular risk factor than systolic hypertension up to age 50 years. Above this age threshold systolic blood pressure is the more potent risk factor [34]. An important and novel additional finding from this study was that in patients aged over 65 years, preoperative diastolic hypotension was associated with a risk of postoperative death and that this association persisted for an adjustment for other risk factors. This finding is biologically plausible in that diastolic pressure is one of the key determinants of mean arterial pressure which in turn is related to organ perfusion. The importance of these findings in the broader context of preoperative assessment is an area of active debate and is potentially relevant to intraoperative management.

## The Intraoperative Management of the Hypertensive Patient

Whilst the primary focus of this review is the preoperative management of the hypertensive surgical patient, this inevitably has a bearing on intraoperative care. It has been noted above and is highlighted in the most recent European guidelines that hypertensive patients may display greater cardiovascular lability during surgery with increases in blood pressure of 20 to 30 mmHg and heart rate of 15 to 20 bpm at induction of anaesthesia and a risk of hypotension in the intraoperative period [35]. A number of large observational studies have now demonstrated an association between intraoperative hypotension and adverse outcome. A study of over 33,000 patients showed an association between a mean arterial pressure less than 55 mmHg and myocardial and renal injury with a linear relationship between the duration of hypotension on the incidence of organ injury [36]. A study of over 24,000 operations conducted in six hospitals showed increased 30-day mortality in patients in the lowest quartile of systolic, mean, and diastolic pressure [37••].

These findings are striking especially when set in the context of the report referenced above an association between preoperative diastolic hypotension and adverse outcome. The concept of autoregulation of organ perfusion is a well-established tenant of physiology [38]. Put simply, the circulation functions as a constant pressure (mean arterial pressure) variable flow system with the flow to individual organs being regulated by arteriolar vascular tone and flow at rest being held constant over a relatively wide range of blood pressure. It has been demonstrated that this autoregulatory range may be shifted to the right (higher blood pressures) in hypertensive individuals. Thus, it may be that patients with hypertension are less tolerant of periods of low blood pressure than their normotensive peers [39]. Randomised controlled trials of the optimal management of intraoperative blood pressure in hypertensive and normotensive patients are lacking but the observational data discussed above and our understanding of cardiovascular physiology suggested that care should be taken to avoid periods of intraoperative hypotension relative to the awake blood pressure, especially in elderly patients.

## Conclusions: Broader Responsibilities

The reader will have gathered from this review that much of the advice and guidance around the pre-and perioperative management of the hypertensive patient are based on epidemiological data rather than randomised controlled trials and that the care of hypertensive individuals requires the combination of an understanding of the evidence including its strengths and limitations and pragmatic clinical judgement on the part of the anaesthetist. It is important to remember that outside of the surgical setting, the evidence for treating raised blood pressure is robust and that doing so can spare the patient the life-changing crisis of a stroke or myocardial infarction. Thus, whilst it may not be appropriate to defer anaesthesia and surgery in a patient with mild or moderate hypertension, the anaesthetist shares a responsibility with other doctors to ensure that the patient found to have persistently elevated blood pressure during preassessment is referred for further blood pressure readings and appropriate management either before or after surgery as appropriate.

## References

[1] Health and Social Care Information Centre. Health Service for England 2011-trend tables. 2011. Available from http://www.hscic.gov.uk.

[2] Whitworth JA (2003). 2003 World Health Organization (WHO)/International Society of Hypertension (ISH) statement on management of hypertension. J Hypertens.

[3] James PA, Oparil S, Carter BL (2014). 2014 evidence-based guideline for the management of high blood pressure in adults: report from the panel members appointed to the Eighth Joint National Committee (JNC 8). JAMA.

[4] Chobanian AV, Bakris GL, Black HR (2003). Seventh report of the joint national Committee on prevention, detection, evaluation, and treatment of high blood pressure. Hypertension.

[5] •• National Institute for Health and Clinical Excellence (NICE). Hypertension: the Clinical Management of Primary Hypertension in Adults: update of Clinical Guidelines 18 and 34. Updated 2016. London: Royal College of Physicians (UK) Available from https://www.nice.org.uk/guidance/cg127. **These updated UK guidelines place particular emphasis on the use of ambulatory monitoring for the diagnosis of hypertension with the implication that isolated blood pressure readings in the hospital setting are of limited value**.

[6] Mancia G, Fagard R, Narkiewicz K, et al. Practice guidelines for the management of arterial hypertension of the European Society of Hypertension (ESH) and the European Society of Cardiology (ESC): ESH/ESC Task Force for the Management of Arterial Hypertension. J Hypertens. 2013;31(7):1925–38. 10.1097/HJH.0b013e328364ca4c 24107724

[7] British Hypertension Society. BP Monitors. 2013. Available from http://www.bhsoc.org/bp-monitors/bp-monitors/.

[8] Staessen JA, O’Brien ET, Amery AK (1994). Ambulatory blood pressure in normotensive and hypertensive subjects: results from an international database. J Hypertens Suppl.

[9] Hamm LL, Hering-Smith KS (2010). Pivotal role of the kidney in hypertension. Am J Med Sci.

[10] Waken RJ, de Las Fuentes L, Rao DC (2017). A review of the genetics of hypertension with a focus on gene-environment interactions. Curr Hypertens Rep.

[11] Laragh JH, Sealey JE (2011). The plasma renin test reveals the contribution of body sodium-volume content (V) and renin-angiotensin (R) vasoconstriction to long-term blood pressure. Am J Hypertens.

[12] Smith SC, Benjamin EJ, Bonow RO (2011). AHA/ACCF secondary prevention and risk reduction therapy for patients with coronary and other atherosclerotic vascular disease: 2011 update: a guideline from the American Heart Association and American College of Cardiology Foundation. Circulation.

[13] National Institute for Health and Care Excellence (NICE). Cardiovascular disease: risk assessment and reduction, including lipid modification. Clinical Guideline (CG181). Updated 2016. Available from https://www.nice.org.uk/guidance/cg181. 36952507

[14] Ahluwalia M, Bangalore S (2017). Management of hypertension in 2017: targets and therapies. Curr Opin Cardiol.

[15] Jackson RE, Bellamy MC (2015). Antihypertensive drugs. Contin Educ Anaesth Crit Care Pain.

[16] • Roshanov PS, Rochwerg B, Patel A, et al. Withholding versus continuing angiotensin-converting enzyme inhibitors or angiotensin II receptor blockers before noncardiac surgery: an analysis of the vascular events in noncardiac surgery patients cohort evaluation prospective cohort. Anesthesiology. 2017;126:16–27. **This secondary analysis of a large observational study includes over 14,000 patients and suggests that angiotensin-converting enzyme inhibitors or angiotensin II receptor blockers may be associated with lower mortality**. 10.1097/ALN.0000000000001404 27775997

[17] Kheterpal S, Khodaparast O, Shanks A, O’Reilly M, Tremper KK (2008). Chronic angiotensin-converting enzyme inhibitor or angiotensin receptor blocker therapy combined with diuretic therapy is associated with increased episodes of hypotension in noncardiac surgery. J Cardiothorac Vasc Anesth.

[18] Twersky RS, Goel V, Narayan P, Weedon J (2014). The risk of hypertension after preoperative discontinuation of angiotensin-converting enzyme inhibitors or angiotensin receptor antagonists in ambulatory and same-day admission patients. Anesth Analg.

[19] Auron M, Harte B, Kumar A, Michota F (2011). Renin-angiotensin system antagonists in the perioperative setting: clinical consequences and recommendations for practice. Postgrad Med J.

[20] Grossman E, Messerli FH, Grodzicki T, Kowey P (1996). Should a moratorium be placed on sublingual nifedipine capsules given for hypertensive emergencies and pseudoemergencies?. JAMA.

[21] Sprague HB (1929). The heart in surgery. An analysis of the results of surgery on cardiac patients during the past ten years at the Massachusetts General Hospital. Surg Gynecol Obstet.

[22] Prys-Roberts C, Foex P, Biro GP, Roberts JG (1973). Studies of anaesthesia in relation to hypertension. V. Adrenergic beta-receptor blockade. Br J Anaesth.

[23] Prys-Roberts C, Greene LT, Meloche R, Foex P (1971). Studies of anaesthesia in relation to hypertension. II. Haemodynamic consequences of induction and endotracheal intubation. Br J Anaesth.

[24] Prys-Roberts C, Meloche R, Foex P (1971). Studies of anaesthesia in relation to hypertension. I. Cardiovascular responses of treated and untreated patients. Br J Anaesth.

[25] Howell SJ, Sear JW, Foex P (2004). Hypertension, hypertensive heart disease and perioperative cardiac risk. Br J Anaesth.

[26] Howell SJ, Sear YM, Sear JW, Yeates D, Goldacre M, Foex P (1997). Nested case-control study of risk factors for cardiovascular death after anaesthesia and surgery. Br J Anaesth.

[27] Howell SJ, Sear YM, Yeates D, Goldacre M, Sear JW, Foex P (1996). Hypertension, admission blood pressure and perioperative cardiovascular risk. Anaesthesia.

[28] Howell SJ, Sear YM, Yeates D, Goldacre M, Sear JW, Foex P (1998). Risk factors for cardiovascular death after elective surgery under general anaesthesia. Br J Anaesth.

[29] Lee TH, Marcantonio ER, Mangione CM (1999). Derivation and prospective validation of a simple index for prediction of cardiac risk of major noncardiac surgery. Circulation.

[30] Fleisher LA, Fleischmann KE, Auerbach AD (2014). 2014 ACC/AHA guideline on perioperative cardiovascular evaluation and management of patients undergoing noncardiac surgery: a report of the American College of Cardiology/American Heart Association Task Force on practice guidelines. J Am Coll Cardiol.

[31] Kristensen SD, Knuuti J, Saraste A (2014). 2014 ESC/ESA guidelines on non-cardiac surgery: cardiovascular assessment and management: the Joint Task Force on non-cardiac surgery: cardiovascular assessment and management of the European Society of Cardiology (ESC) and the European Society of Anaesthesiology (ESA). Eur Heart J.

[32] Howell SJ, Hemming AE, Allman KG, Glover L, Sear JW, Foex P (1997). Predictors of postoperative myocardial ischaemia. The role of intercurrent arterial hypertension and other cardiovascular risk factors. Anaesthesia.

[33] Venkatesan S, Myles P, Manning H (2017). Cohort study evaluating preoperative blood pressure (BP) values and risk of 30-day mortality following elective non-cardiac surgery. Br J Anaesth.

[34] Franklin SS, Larson MG, Khan SA (2001). Does the relation of blood pressure to coronary heart disease risk change with aging? The Framingham Heart Study. Circulation.

[35] Kihara S, Brimacombe J, Yaguchi Y, Watanabe S, Taguchi N, Komatsuzaki T (2003). Hemodynamic responses among three tracheal intubation devices in normotensive and hypertensive patients. Anesth Analg.

[36] Walsh M, Devereaux PJ, Garg AX (2013). Relationship between intraoperative mean arterial pressure and clinical outcomes after noncardiac surgery: toward an empirical definition of hypotension. Anesthesiology.

[37] •• Monk TG, Bronsert MR, Henderson WG, et al. Association between intraoperative hypotension and hypertension and 30-day postoperative mortality in noncardiac surgery. Anesthesiology. 2015;123:307–19. **This large observational study suggests that intraopertive hypotension is associated with increased 30-day postoperative mortality**. 10.1097/ALN.0000000000000756 26083768

[38] Lassen NA (1964). Autoregulation of cerebral blood flow. Circ Res.

[39] Ruland S, Aiyagari V (2007). Cerebral autoregulation and blood pressure lowering. Hypertension.

